# Biomechanical evaluation of four internal fixation systems for two-segment anterior cervical corpectomy and fusion: a finite element analysis

**DOI:** 10.3389/fbioe.2025.1691524

**Published:** 2025-10-28

**Authors:** Wei Yuan, Lei Pei, Han Wang, Yicheng Liu, Ruohan Kang, Yue Zhu

**Affiliations:** Department of Orthopedics, The First Hospital of China Medical University, Shenyang, China

**Keywords:** cervical spine, internal fixation, transpedicular screw, artificial vertebral body, finiteelement analysis

## Abstract

**Background:**

Anterior cervical corpectomy and fusion (ACCF) is a key surgical intervention for cervical spine pathologies, but multi-segment ACCF is associated with high risks of instability, implant failure, and adjacent segment degeneration (ASD). Conventional internal fixation system, vertebral body screw (VBS), titanium plate (TP), combine with titanium mesh cage (TMC) have limitations such as insufficient three-column fixation. Anterior transpedicular screws (ATPS) and 3D-printed artificial vertebral bodies (3D-AVB) have shown potential to improve biomechanical performance, but direct biomechanical comparisons of different internal fixation system systems in two-segment ACCF remain lacking.

**Methods:**

A finite element (FE) model of the C1-T1 cervical spine was constructed and validated. Four two-segment ACCF (C5-C6 corpectomy) models with different internal fixation systems were established: (1) Group 1: four VBS with TP and TMC; (2) Group 2: four oblique VBS with 3D-AVB; (3) Group 3: two superior VBS and two inferior ATPS with 3D-AVB; (4) Group 4: four ATPS with 3D-AVB. A 73.6 N axial load and 1.0 Nm moment were applied. Outcome measures included range of motion (ROM) of surgical (C4-C7) and adjacent segments, maximum von Mises stress of implants, bone-implant interfaces, and adjacent intervertebral discs.

**Results:**

All four groups reduced C4-C7 ROM, with Group 4 showing the most significant reduction (≈98% for flexion/extension), followed by Group 3, Group 2, and Group 1 (68.5% flexion reduction). Group 4 exhibited slightly increased adjacent segment ROM (54.8% increase in C3-C4 extension). Implant stress was lowest in Group 4 (54.8 MPa) and highest in Group 2 (255.8 MPa). Group 4 also had the lowest bone-implant interface stress at C4 and T1, whereas Group 2 had the highest. Adjacent disc stress in Group 4 was comparable to the intact model, while other groups showed increases (46.9% in Group 1).

**Conclusion:**

The Group 4 (4 ATPS + 3D-AVB system) is preferred for two-segment ACCF, as it provides superior surgical segment stability, reduces stress on implants, bone-implant interfaces, and adjacent discs. For cases where Group 4 is inapplicable, alternatives should be prioritized as follows: Group 3 (2 VBS +2 ATPS + 3D-AVB), Group 2 (4 oblique VBS + 3D-AVB), and Group 1 (4 VBS + TP + TMC). Long-term efficacy of these systems requires verification via future clinical trials.

## 1 Introduction

Anterior cervical corpectomy and fusion (ACCF) emerged in the 1960s, initially used for treating cervical spine fractures (Foreman et al., 2024). Over time, its application has gradually expanded to cover various cervical pathologies—particularly those with pathogenic sites at the vertebral body level, where anterior cervical discectomy and fusion (ACDF) is considered unsuitable (Verma et al., 2025). Such pathologies include degenerative, traumatic, neoplastic, and infectious conditions affecting the cervical spine.

In the context of single-segment ACCF, favorable patient outcomes have long been documented (Xiao et al., 2015). However, multiple studies have highlighted a high risk of early implant failure and instability following multi-segment ACCF (Singh et al., 2003; Bayerl et al., 2019). Specifically, multi-segment cervical decompression and reconstruction often fail to achieve adequate stability with ACCF alone; literature indicates that instrumentation-related complications in multi-segment ACCF are common, occurring in up to 75% of cases and frequently necessitating revision surgery (Sasso et al., 2003; Wang et al., 2003; Daubs, 2005). Some researchers have recommended supplementary posterior spinal fixation and fusion after multi-segment ACCF to enhance the stability of anterior fixation and reduce surgical failure rates (Oni et al., 2018; Bayerl et al., 2019). For selected patients with complex cervical spinal disorders, the single-stage combined anterior-posterior decompression, reconstruction, and instrumentation procedure represents a viable option. This technique provides immediate rigid stabilization of the cervical spine, preventing anterior plate failure or strut graft extrusion (Schultz et al., 2000). Nevertheless, combined anterior and posterior surgery not only increases the economic burden but also prolongs operative time and elevates the risks of iatrogenic trauma and complications (Wewel et al., 2020). Thus, if anterior cervical implants could provide more effective stabilization, patients could avoid the longer operative duration and heightened complication risks associated with the additional posterior approach.

With advancements in internal fixation techniques, vertebral body screws (VBS), titanium plates (TP), and titanium mesh cages (TMC) have become the mainstream internal fixation system for ACCF. This system can restore the physiological height of the vertebral body and provide immediate, robust anterior column support (Dorai et al., 2003). However, VBS only achieves unicortical fixation of the anterior and middle columns, failing to provide effective three-column stabilization. Additionally, TMC does not match the morphology of the vertebral endplate, leading to partial contact that is prone to stress concentration—a factor associated with the reported moderate subsidence rates (Ji et al., 2020). Furthermore, long strut grafts and plate fixations create long lever arms that exert excessive stress on the caudal screws, making patients susceptible to graft migration, displacement, and instrumentation failure.

To enhance the stability of anterior screw fixation, the anterior transpedicular screw (ATPS) technique was first introduced into ACCF by Aramomi et al. (2008). Subsequent biomechanical studies by Koller et al. (Koller et al., 2008a; Koller et al., 2008b; Koller et al., 2010) identified key advantages of ATPS: The pull-out strength of ATPS reached 2.5 times that of VBS, mainly attributed to its effective engagement with the dense cortical bone of cervical pedicles, its implantation in the pedicle region (which has significantly higher BMD than the anterior vertebral body), and a notably longer osseous screw purchase length relative to VBS, and the stability provided by the ATPS-TP-TMC fixation system was nearly comparable to that of 360° spinal reconstruction. However, inaccurate placement of ATPS carries a high risk of complications, such as vertebral artery injury and nerve damage, and there is currently no widely accepted ATPS-plate system for clinical use, this has limited the widespread clinical application of cervical ATPS fixation. In addition, 3D-printed artificial vertebral body (3D-AVB) have been reported, characterized by a morphology matching the vertebral endplate and a porous structure. Theoretically, they offer advantages such as improved load distribution, enhanced osseointegration, reduced stress shielding, and a potential lower risk of subsidence (Hunn et al., 2020; Pei et al., 2022), making them a promising future alternative to TMC. Moreover, 3D-AVB can be designed with screw holes to accommodate bilateral ATPS insertion (Pei et al., 2022). While these technologies provide more internal fixation options for ACCF, there remains a lack of direct biomechanical research on these internal fixation systems in the context of ACCF.

The finite element (FE) method is an ideal tool for studying spinal biomechanics. It offers the advantage of predicting the cervical biomechanical response to different cervical prosthesis designs; as a complement to animal and cadaver studies, it has been widely used (Tchako and Sadegh, 2009; Yuan et al., 2018; Ye et al., 2024). This study aims to explore the biomechanical properties of four distinct internal fixation systems for two-segment ACCF via FE analysis, specifically: (1) two superior and two inferior VBS with TP and TMC; (2) two superior and two inferior VBS with a 3D-AVB; (3) two superior VBS and two inferior ATPS with a 3D-AVB; (4) two superior and two inferior ATPS with a 3D-AVB. By analyzing range of motion (ROM), implant stress, bone-implant interface stress, and adjacent intervertebral disc stress, this study seeks to provide biomechanical references for clinical selection of internal fixation systems in two-segment ACCF.

## 2 Materials and methods

### 2.1 Subjects

A 38-year-old healthy adult female volunteer (height: 168 cm; weight: 56 kg) with no history of cervical spine disease was enrolled. Cervical radiographs (anteroposterior, lateral, oblique, hyperextension, and hyperflexion views) were obtained to rule out pathological conditions. This study was approved by the Ethics Committee of our hospital (No. 2024-674-2). Additionally, as the study was based solely on imaging data, it posed no harm to the volunteer and ensured the protection of their personal information.

### 2.2 Establishment of the intact FE model

**FIGURE 1:**
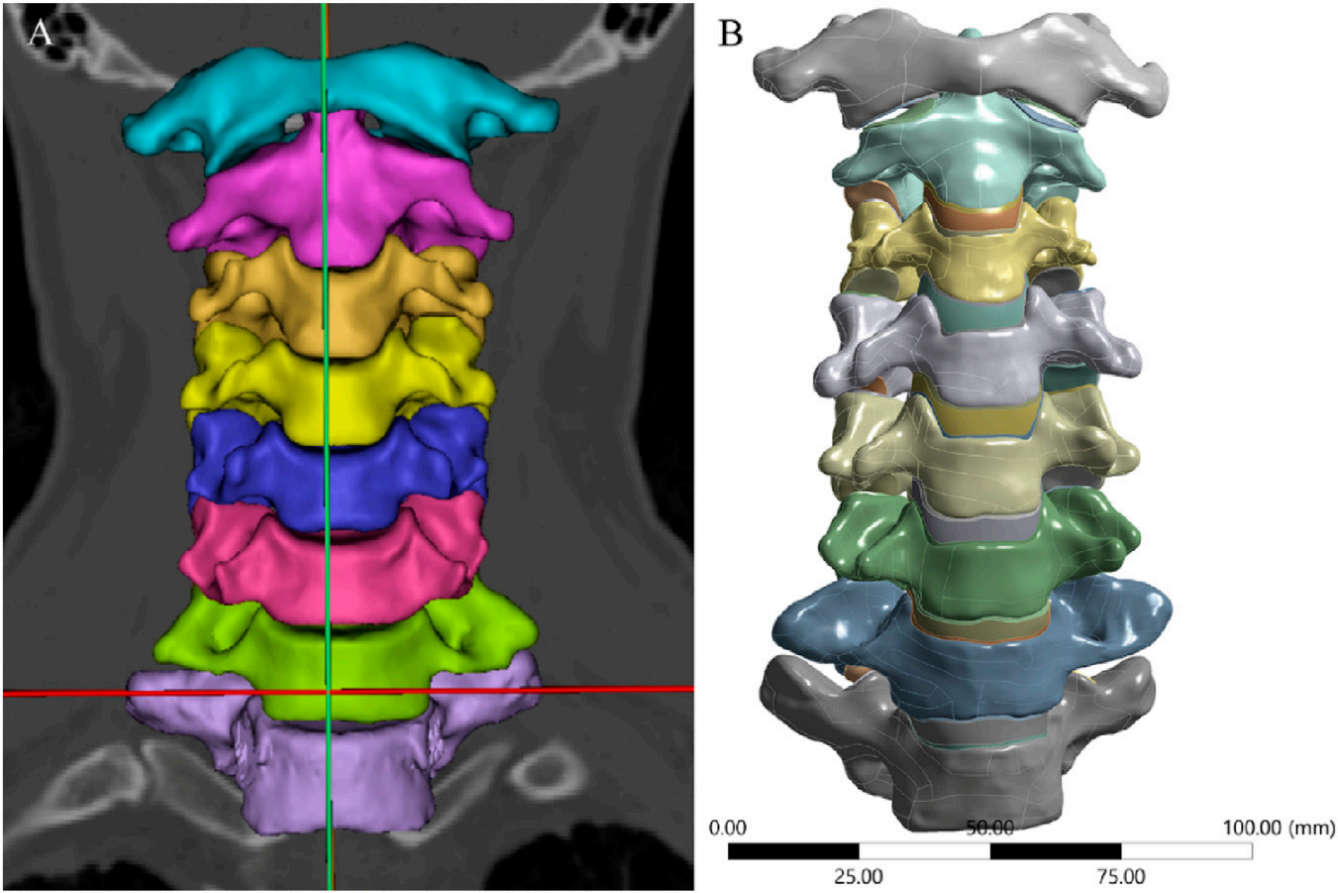
3D Model of the Intact Cervical Spine. **(A)** 3D geometric data reconstructed using Mimics, **(B)** Surface refinement and optimization of the model performed via Geomagic Studio.

Cervical spine images were acquired using a GE 64-slice scanner (GE Healthcare, United States) with a slice thickness of 0.625 mm. The images were saved in DICOM format and imported into Mimics 21.0 (Materialise, Belgium). After segmenting the bony structures, three-dimensional graphic data in stereolithography (STL) format (Figure 1A) were imported into Geomagic Studio 2021 (3D Systems, United States) for surface refinement and optimization. Cortical and cancellous bones were reconstructed separately to achieve anatomically accurate modeling, and the model was exported in STEP format (Figure 1B). The STEP file was then imported into ANSYS Workbench 2024 (ANSYS, United States) for FE preprocessing. Using SpaceClaim, intervertebral discs, endplate cartilage, nucleus pulposus, and articular cartilage were reconstructed via Extrude/Loft operations (based on CT anatomical morphology), resulting in a multi-body C1-T1 model with shared topology. Intervertebral discs were curved-surface modeled using CT data contours. To avoid disc-endplate gaps, the upper and lower parts of each disc were first over-modeled, with excess material later removed via Boolean operations. Disc matrix-nucleus pulposus differentiation referenced Mercer’s study (Mercer and Bogduk, 1999). For annulus fibrosus fibers, APDL language was added to each disc model in ANSYS Workbench’s Model Design module; fibers were formed by linking nodes on the annulus fibrosus matrix and simulated using uniaxial tension Combin39 elements. Based on the anatomical characteristics of the cervical intervertebral disc and previous studies (Xue et al., 2021), we set its thickness to 4 mm. The nucleus pulposus accounts for approximately 34% of the total intervertebral disc volume.

**FIGURE 2:**
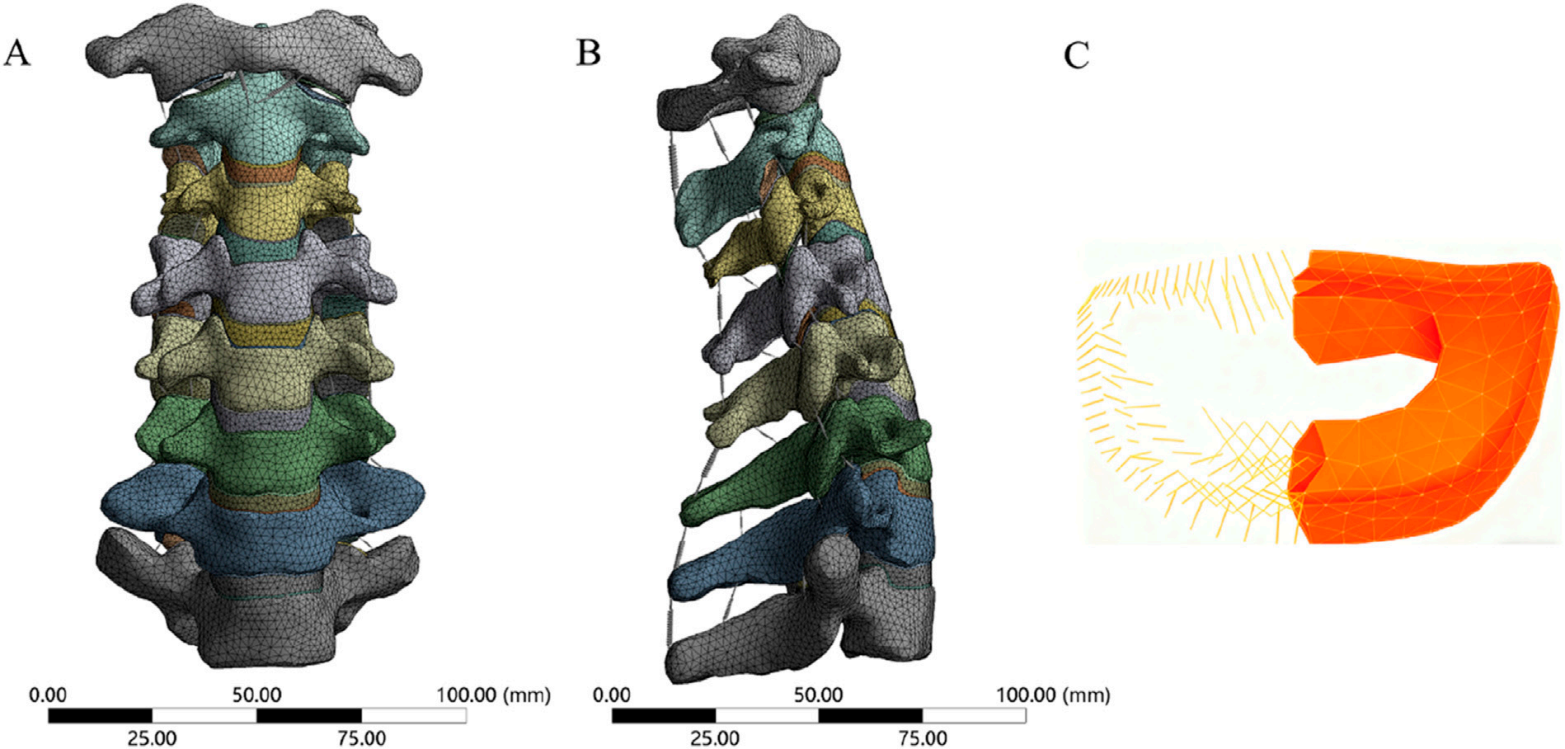
The FE model of cervical spine. **(A)** anterior view, **(B)** lateral view, **(C)** Annulus matrix and fibers.

We referenced previous studies on cervical spine FE models to determine the material properties for cervical spine reconstruction, including those of cortical bone, cancellous bone, facet cartilage, endplates, nucleus pulposus, annulus matrix, and annulus fibers (Table 1) (Lee et al., 2011; Ye et al., 2024). The anterior longitudinal ligament (ALL), posterior longitudinal ligament (PLL), interspinous ligament (IL), supraspinous ligament (SL), capsular ligament (CL), and ligamentum flavum (LF) are simulated using nonlinear 3D rod elements (Link180), with the working mode set to tension only. (Table 2) (Tchako and Sadegh, 2009; Wu et al., 2017). An intact 3-dimensional FE model of the C3-C7 spinal segment was constructed, featuring a realistic anatomical shape with a total of 1,522,884 elements and 2,610,333 nodes (Figure 2). Mesh quality assessment was made in accordance with Burkhart’s study (Burkhart et al., 2013): Mesh shape: cartilages were meshed with hexahedrons; the other parts were tetrahedrons. Aspect ratio: min 2.36, max 7.81, average 4.22. Angle idealization: min 35.35, max 128.11, average 67.53. Element Jacobians: min 0.43, max 0.99, average 0.68. The mesh size employed in this study is 0.5 mm. Validation was verified by measuring the range of motion (ROM) of each vertebra and comparing them with the *in vitro* biomechanical test and previous finite element analysis results.

**TABLE 1:** Material properties of finite element model.

	Element type	Young’s modulus (MPa)	Poisson’s ratio
Vertebra			
Cortical	solid (Isotropic)	12 000	0.29
Cancellous	solid (Isotropic)	450	0.29
Facet cartilage	solid (Isotropic)	10.4	0.4
Endplate	solid (Isotropic)	500	0.4
Disc			
Nucleus pulposus	solid (Isotropic)	1	0.499
Annulus matrix	solid (Isotropic)	3.4	0.4
Annulus fibers	link (Two-node)	110	0.3
Titanium alloy implant	solid (Isotropic)	110 000	0.3

**TABLE 2:** Ligaments tensile properties of cervical spine.

ALL		PLL		IS		LF		CL	
Deflexion	Force	Deflexion	Force	Deflexion	Force	Deflexion	Force	Deflexion	Force
(mm)	(N)	(mm)	(N)	(mm)	(N)	(mm)	(N)	(mm)	(N)
0	0	0	0	0	0	0	0	0	0
1.4	12	1	9.65	1.3	4.2	1.9	6.7	1.8	6.7
2.7	18	2	17.15	2.7	6.1	3.9	11	3.9	11
4.1	22.5	3	23.76	4	7.4	5.8	13.7	5.8	13.7
5.4	27.15	4	28.6	5.4	8.2	7.7	15.7	7.7	15.7
6.8	30	5	31.6	6.7	8.8	9.7	16.85	9.7	16.85

ALL, anterior longitudinal ligament; PLL, posterior longitudinal ligament; IS, interspinal ligament; LF, ligamentum flavum; CL, capsular ligament.

### 2.3 Establishment of two-segment ACCF models with four different internal fixation systems

**FIGURE 3:**
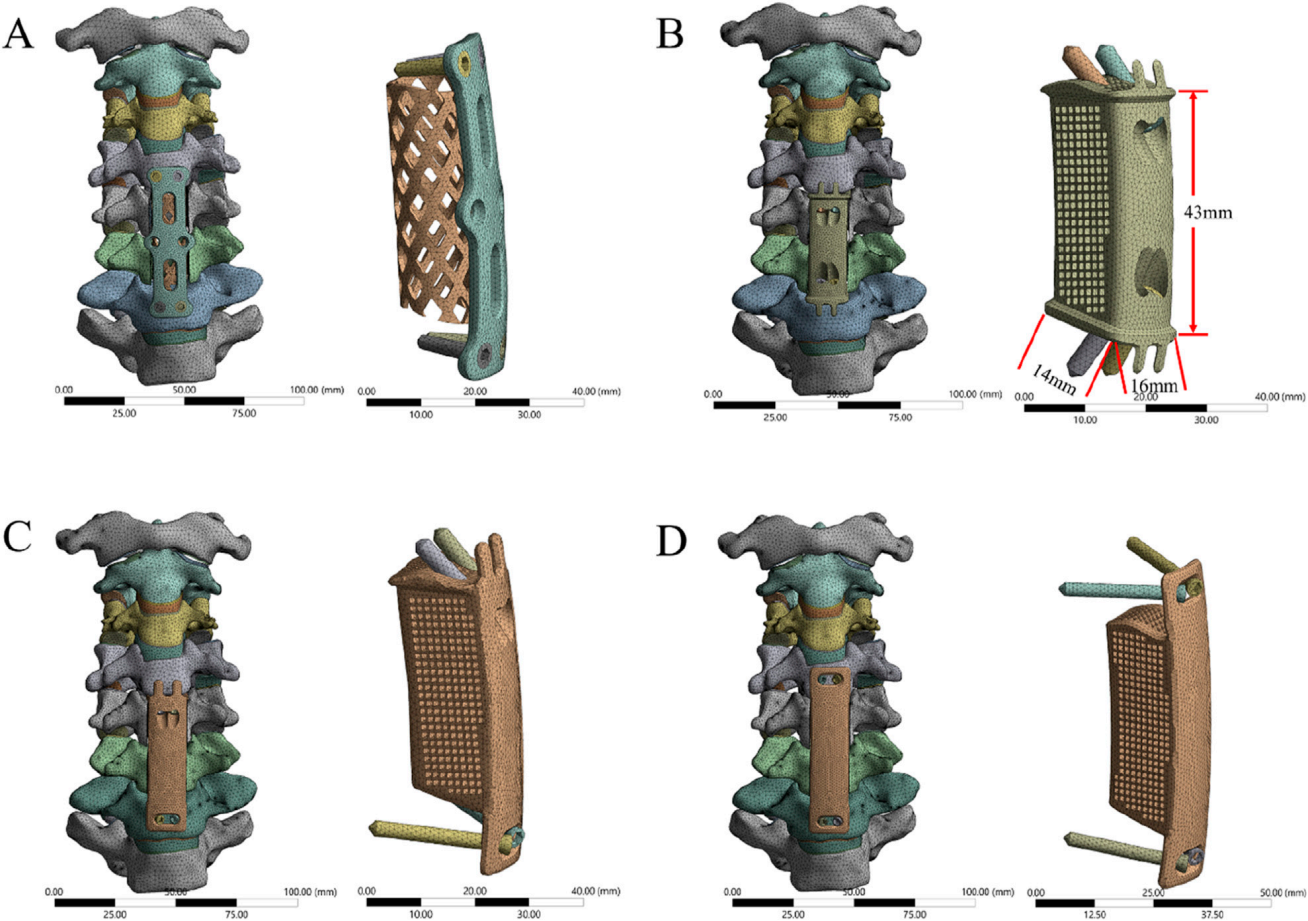
FE models of two-segment ACCF with four different internal fixation systems. **(A)** Four VBS combined with TP and TMC, **(B)** Four VBS combined with a 3D-AVB, **(C)** Two superior VBS, two inferior ATPS, and a 3D-AVB, **(D)** Four ATPS combined with a 3D-AVB.

First, the two-segment ACCF model was simulated. Two-segment corpectomies were performed by resecting the C5 and C6 vertebral bodies along with their adjacent intervertebral discs; additionally, the ALL and PLL from the C4-C7 segment were removed. Subsequently, a TMC or AVB was centrally placed between the inferior endplate of C4 and the superior endplate of C7. Four experimental groups were established (Figure 3), as follows: (1) Group 1: Four VBS combined with TP and TMC; (2) Group 2: Four VBS combined with a 3D-AVB; (3) Group 3: Two superior VBS, two inferior ATPS, and a 3D-AVB; (4) Group 4: Four ATPS combined with a 3D-AVB.

### 2.4 Design of 3D-AVB

Using 3D reconstruction data from preoperative CT images of the surgical segment, the vertebral endplate morphology was accurately reproduced to enable surface contact between the 3D-AVB and the C4 inferior endplate/C7 superior endplate—this eliminates stress concentration caused by the point contact of traditional titanium mesh cages. The 3D-AVB features a porous structure (porosity: 60%; pore size: 500–800 μm) that balances mechanical strength with channels for osteocyte migration and vascular ingrowth, facilitating osseointegration. Four preset screw holes in the 3D-AVB (aligned with cervical pedicle/vertebral body anatomy) support both ATPS and VBS implantation. Screw dimensions are 3.5 mm × 15 mm (VBS) and 3.5 mm × 35 mm (ATPS).

### 2.5 Loading and boundary conditions

Constraints were applied to the FE models, with the lower endplate of T1 fully fixed and C1 left unconstrained. Referring to previous literature, an axial load of 73.6 N was applied to the superior surface of the C1 to simulate the weight of the head, while a 1.0 Nm moment was applied at the coupling point on the superior surface of C1 to induce anterior flexion, posterior extension, lateral flexion, and axial rotation in the FE models (Panjabi et al., 2001; Wu et al., 2017). Facet joints were defined as frictionless surface-to-surface contact. Screws and internal fixation devices were bonded to simulate a locked state, whereas titanium meshes/plates and vertebral bodies were assigned frictional contact (friction coefficient: 0.65) (Wu et al., 2017).

### 2.6 Evaluation indicators

For each model, the following parameters were recorded and comparatively analyzed: the ROM of the surgical segment and adjacent segments, the maximum von Mises stress on the internal fixation system, the maximum von Mises stress at the bone-implant interface, and the maximum von Mises stress on the adjacent intervertebral discs.

## 3 Results

### 3.1 Model validation

The intact cervical spine FE model used in this study was validated in our previous work (Wu et al., 2017; Yuan et al., 2018). For the current intact cervical spine FE model, the ranges of motion (ROM) at the C3-C4, C4-C5, C5-C6, and C6-C7 segments were as follows: during flexion: 5.6°, 6.0°, 5.2°, and 3.4°, respectively; during extension: 3.1°, 6.4°, 5.8°, and 5.1°, respectively; during left lateral bending: 11.7°, 10.4°, 9.7°, and 5.5°, respectively; during right lateral bending: 12.1°, 10.4°, 9.7°, and 5.5°, respectively; during left axial rotation: 8.6°, 7.8°, 5.1°, and 4.6°, respectively; during right axial rotation: 8.3°, 7.7°, 5.0°, and 4.9°, respectively. The range of motion (ROM) values of the current model showed high consistency with those reported in previous studies (Panjabi et al., 2001; Ye et al., 2024), as presented in Supplementary Figure S1. This alignment confirms the validity of the model established in the present study.

### 3.2 ROMs of the surgical segment and adjacent segments

**FIGURE 4:**
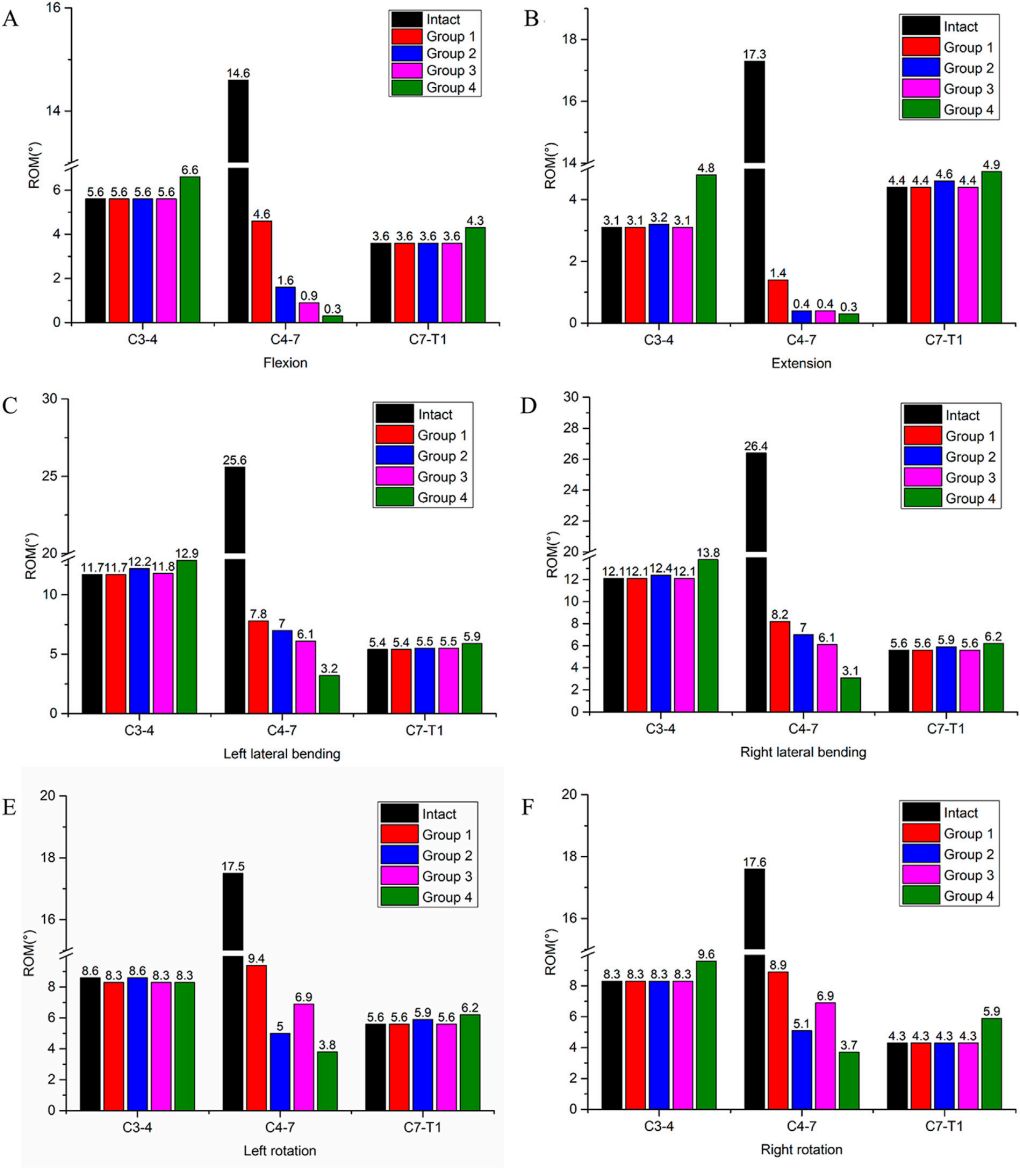
ROM of the surgical segment (C4-C7) and adjacent segments (C3-C4, C7-T1) in the intact model and four different internal fixation models. **(A)** ROM in flexion, **(B)** ROM in extension, **(C)** ROM in left lateral bending, **(D)** ROM in right lateral bending, **(E)** ROM in left rotation, **(F)** ROM in right rotation.

The ROM of each segment is presented in Table 3. For the intact surgical segments (C4-C7), the ROM in flexion, extension, left lateral bending, right lateral bending, left rotation, and right rotation were measured as 14.6°, 17.3°, 25.6°, 26.4°, 17.5°, and 17.6°, respectively. After the two-segment ACCF procedure, all four internal fixation methods significantly reduced the ROM of the C4-C7 segments (Figure 4). Specifically, Group 4 exhibited the greatest reduction in ROM across all six motion directions compared to the other groups. Among these six directions, all groups showed the strongest restriction on extension movement, followed by flexion. The specific ROM reduction rates of the C4-C7 segments in each group are as follows: Group 4: 97.9% reduction in flexion, 98.3% in extension, 87.5% in left lateral bending, 88.3% in right lateral bending, 78.3% in left rotation, and 78.9% in right rotation; Group 3: 93.2% reduction in flexion, 98.2% in extension, 76.1% in left lateral bending, 76.9% in right lateral bending, 60.6% in left rotation, and 60.8% in right rotation; Group 2: 89.0% reduction in flexion, 98.2% in extension, 72.7% in left lateral bending, 73.5% in right lateral bending, 71.4% in left rotation, and 71.0% in right rotation; Group 1: 68.5% reduction in flexion, 98.2% in extension, 69.5% in left lateral bending, 68.9% in right lateral bending, 46.3% in left rotation, and 49.4% in right rotation.

**TABLE 3:** ROM of the surgical segment and adjacent segments.

	Intact (°)	Group 1 (°)	Group 2 (°)	Group 3 (°)	Group 4 (°)
Flexion					
C3-4	5.6	5.6	5.6	5.6	6.6
C4-5	6	1.5	0.6	0.6	0.1
C5-6	5.2	1.6	0.6	0.2	0.1
C6-7	3.4	1.5	0.4	0.1	0.1
C7-T1	3.6	3.6	3.6	3.6	4.3
Extension					
C3-4	3.1	3.1	3.2	3.1	4.8
C4-5	6.4	0.5	0.1	0.2	0.1
C5-6	5.8	0.5	0.2	0.1	0.1
C6-7	5.1	0.4	0.1	0.1	0.1
C7-T1	4.4	4.4	4.6	4.4	4.9
Left lateral bending					
C3-4	11.7	11.7	12.2	11.8	12.9
C4-5	10.4	3.6	3.1	3.1	1
C5-6	9.7	1.9	2.3	1.9	1.1
C6-7	5.5	2.3	1.6	1.1	1.1
C7-T1	5.4	5.4	5.5	5.5	5.9
Right lateral bending					
C3-4	12.1	12.1	12.4	12.1	13.8
C4-5	10.7	3.5	3.2	3.3	1.1
C5-6	9.9	2.1	2.3	1.8	0.9
C6-7	5.8	2.6	1.5	1	1.1
C7-T1	5.6	5.6	5.9	5.6	6.2
Left rotation					
C3-4	8.6	8.3	8.6	8.3	8.3
C4-5	7.8	3.9	1.7	3.1	1.2
C5-6	5.1	2.7	1.5	2.1	0.9
C6-7	4.6	2.8	1.8	1.7	1.7
C7-T1	4.2	4.3	4.3	4.3	4.3
Right rotation					
C3-4	8.3	8.3	8.3	8.3	9.6
C4-5	7.7	3.7	1.7	3.2	1.3
C5-6	5	2.6	1.5	2.1	0.9
C6-7	4.9	2.6	1.9	1.6	1.5
C7-T1	4.3	4.3	4.3	4.3	5.9

Regarding the ROM of adjacent segments, an increase in ROM was observed only in Group 4. Specifically, in flexion, the ROM of the C3-C4 segment increased by 17.9%, and that of the C7-T1 segment increased by 19.4%; in extension, the ROM of the C3-C4 segment increased by 54.8%, and that of the C7-T1 segment increased by 11.4%. This phenomenon was not detected in the other three groups.

### 3.3 Stress on the internal fixation system

Under the applied loading conditions, Group 4 exhibited relatively lower maximum von Mises stress compared to the other groups. Specifically, the maximum von Mises stress values for Group 4 during flexion, extension, left lateral bending, right lateral bending, left rotation, and right rotation were 35.0 MPa, 33.9 MPa, 54.8 MPa, 54.5 MPa, 28.8 MPa, and 28.4 MPa, respectively. In contrast, Groups 1, 2, and 3 showed the highest stress values in the flexion and extension directions among all six motion directions. The specific maximum von Mises stress values for these groups in flexion and extension were as follows: Group 1: 180.4 MPa (flexion) and 126.5 MPa (extension); Group 2: 255.8 MPa (flexion) and 254.7 MPa (extension); Group 3: 113.5 MPa (flexion) and 113.6 MPa (extension) (Table 4). The highest maximum von Mises stress in Group 4 was 54.8 MPa. Compared with the peak stress values of the other groups, this represented a reduction of: 69.6% relative to Group 1 (180.4 MPa); 78.6% relative to Group 2 (255.8 MPa); 51.7% relative to Group 3 (113.5 MPa).

**TABLE 4:** The maximum von Mises stress on internal fixation system.

	Group 1 (MPa)	Group 2 (MPa)	Group 3 (MPa)	Group 4 (MPa)
Flexion	180.4	255.8	113.5	35.0
Extension	126.5	254.7	113.6	33.9
Left lateral bending	78.0	110.0	30.1	54.8
Right lateral bending	79.1	109.4	29.7	54.5
Left rotation	128.8	142.6	59.6	28.8
Right rotation	129.7	143.4	58.9	28.4

**FIGURE 5:**
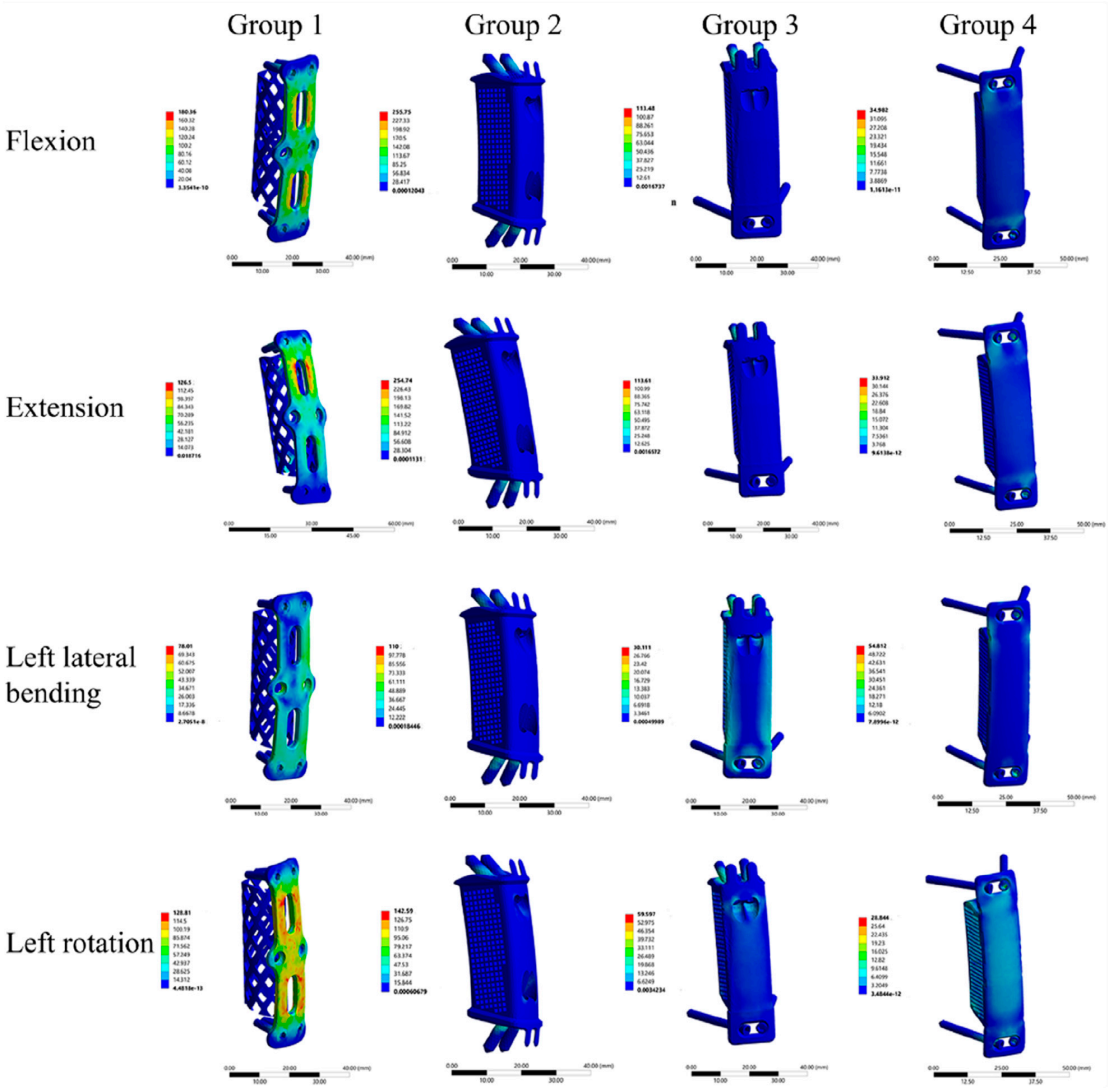
Stress cloud maps of the four internal fixation systems under flexion, extension, lateral bending, and axial rotation conditions.

Stress cloud maps of the internal fixation system for the four groups under flexion, extension, lateral bending, and axial rotation conditions are presented in Figure 5. It was observed that the maximum von Mises stress was predominantly concentrated at the screws or the plate.

### 3.4 Stress at bone-implant interface

**FIGURE 6:**
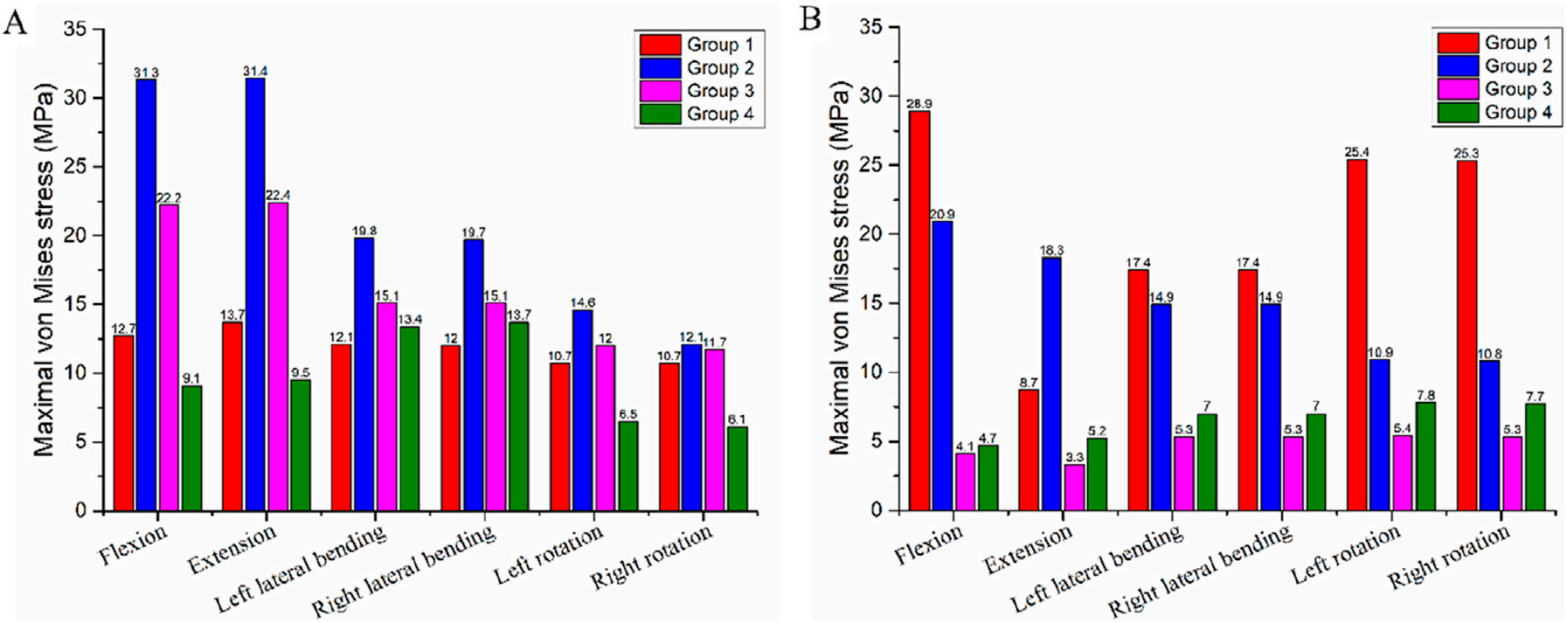
Maximum von Mises stress at the bone-implant interface in the four different internal fixation models. **(A)** Stress at the C4-implant interface, **(B)** Stress at the T1-implant interface.

At the C4-implant interface, Group 4 exhibited the lowest von Mises stress across all motion directions. Specifically, the stress values for Group 4 during flexion, extension, left lateral bending, right lateral bending, left rotation, and right rotation were 9.1 MPa, 9.5 MPa, 13.4 MPa, 13.7 MPa, 6.5 MPa, and 6.1 MPa, respectively (Table 5). In contrast, Group 2 showed the highest von Mises stress among the four groups (31.3 MPa, 31.4 MPa, 19.8 MPa, 19.7 MPa, 14.6 MPa, and 12.1 MPa, respectively), followed by Group 3 and then Group 1 (Figure 6). The maximum stress at the C4-implant interface in Group 4 was 13.7 MPa. Compared with the maximum stresses of other groups, this represented a: 56.4% reduction relative to Group 2 (31.4 MPa); 38.8% reduction relative to Group 3 (22.4 MPa).

**TABLE 5:** The maximum von Mises stress at bone-implant Interface.

	Group 1 (MPa)	Group 2 (MPa)	Group 3 (MPa)	Group 4 (MPa)
Flexion				
C4	12.7	31.3	22.2	9.1
T1	28.9	20.9	4.1	4.7
Extension				
C4	13.7	31.4	22.4	9.5
T1	8.7	18.3	3.3	5.2
Left lateral bending				
C4	12.1	19.8	15.1	13.4
T1	17.4	14.9	5.3	7.0
Right lateral bending				
C4	12.0	19.7	15.1	13.7
T1	17.4	14.9	5.3	7.0
Left rotation				
C4	10.7	14.6	11.9	6.5
T1	25.4	10.9	5.4	7.8
Right rotation				
C4	10.6	12.1	11.7	6.1
T1	25.3	10.8	5.3	7.7

At the T1-implant interface, Group 3 and Group 4 displayed similar and lower stress values across all six motion directions. The stress values were: Group 3: 4.1 MPa, 3.3 MPa, 5.3 MPa, 5.3 MPa, 5.4 MPa, and 5.3 MPa; Group 4: 4.7 MPa, 5.2 MPa, 7.0 MPa, 7.0 MPa, 7.8 MPa, and 7.7 MPa (Table 5). Group 1 exhibited the highest stress at this interface (28.9 MPa, 8.7 MPa, 17.4 MPa, 17.4 MPa, 25.4 MPa, and 25.3 MPa), followed by Group 2 (Figure 6). The maximum stress at the T1-implant interface in Group 4 was 7.8 MPa. This corresponded to a: 73.0% reduction relative to Group 1 (28.9 MPa); 63.7% reduction relative to Group 2 (20.0 MPa).

**FIGURE 7:**
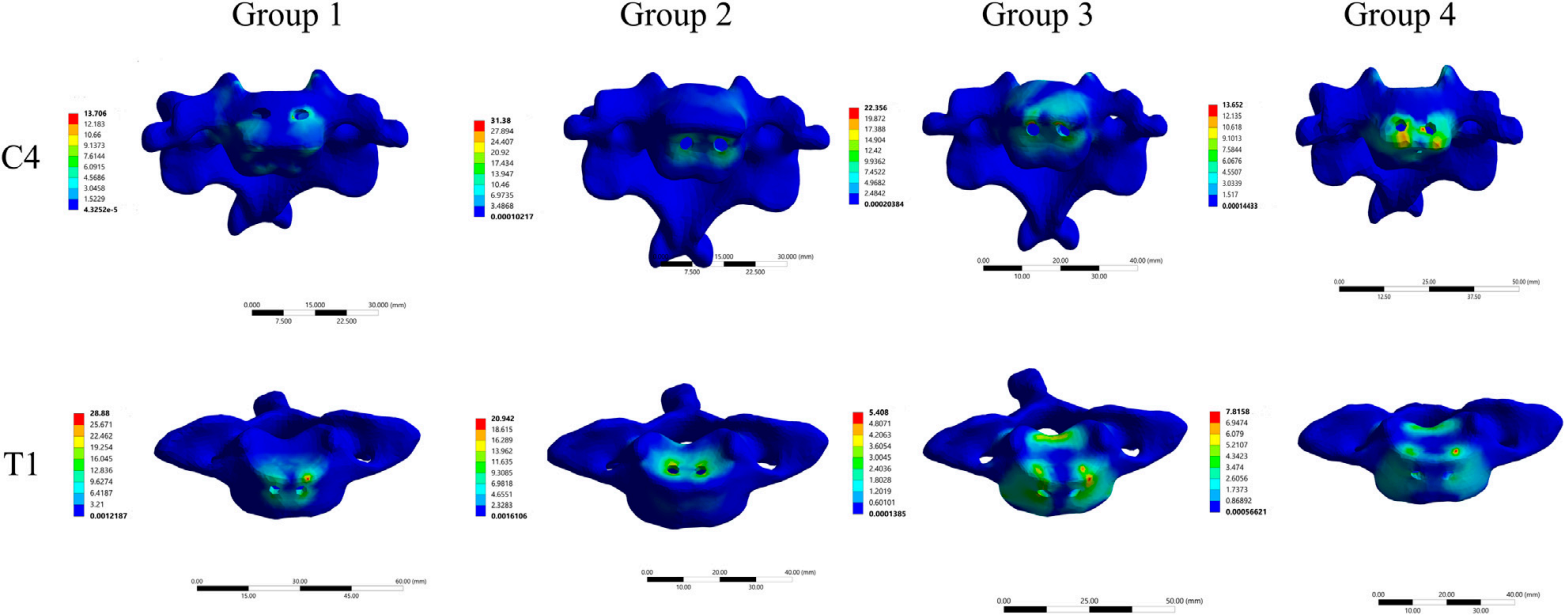
Stress cloud maps of the maximum stress at the C4-implant and T1-implant interfaces in the four different internal fixation models.

Stress cloud maps showing the peak stress values at the C4 and T1 implant interfaces for the four groups are illustrated in Figure 7. It was observed that the maximum von Mises stress was predominantly concentrated at the screw holes in C4, while in T1, the maximum stress was concentrated at both the screw holes and the endplate.

### 3.5 Stress on the intervertebral disc

In the intact model, the maximum von Mises stress values of the C3-C4 disc during flexion, extension, left lateral bending, right lateral bending, left rotation, and right rotation were 2.8 MPa, 3.6 MPa, 3.2 MPa, 3.0 MPa, 5.2 MPa, and 3.2 MPa, respectively (Table 6). For Group 4, the von Mises stress values of the C3-C4 disc were 3.1 MPa, 3.8 MPa, 3.5 MPa, 3.2 MPa, 4.3 MPa, and 3.4 MPa, these values were similar to those in the intact model. Compared with Groups 1, 2, and 3, Group 4 had the minimal impact on the stress of the adjacent-segment intervertebral disc. Specifically, relative to the intact model: Group 1 showed the largest stress increase, at 46.9% (reaching 4.7 MPa); Group 2 showed a maximum stress increase of 10.7% (reaching 4.1 MPa); Group 3 showed a maximum stress increase of 28.1% (reaching 4.1 MPa).

**TABLE 6:** The maximum von Mises stress on the intervertebral disc.

	Intact (MPa)	Group 1 (MPa)	Group 2 (MPa)	Group 3 (MPa)	Group 4 (MPa)
Flexion					
C3-4	2.8	3.4	3.1	4.0	3.1
C7-T1	0.8	0.8	0.9	0.9	0.9
Extension					
C3-4	3.6	4.2	3.8	4.6	3.8
C7-T1	1.2	1.3	1.3	1.4	1.3
Left lateral bending					
C3-4	3.2	4.7	3.4	4.1	3.5
C7-T1	2.4	3.0	2.5	2.4	2.1
Right lateral bending					
C3-4	3.0	4.3	3.2	3.7	3.2
C7-T1	2.6	3.3	2.6	2.6	2.3
Left rotation					
C3-4	5.2	5.1	4.1	4.3	4.3
C7-T1	3.3	4.0	3.5	3.6	3.1
Right rotation					
C3-4	3.2	3.8	3.5	3.7	3.4
C7-T1	3.1	3.7	3.4	3.3	2.8

In the intact model, the maximum von Mises stress values of the C7-T1 disc during flexion, extension, left lateral bending, right lateral bending, left rotation, and right rotation were 0.8 MPa, 1.2 MPa, 2.4 MPa, 2.6 MPa, 3.3 MPa, and 4.3 MPa, respectively (Table 6). For Group 4, the von Mises stress values of the C7-T1 disc were 0.9 MPa, 1.3 MPa, 2.09 MPa, 2.3 MPa, 3.1 MPa, and 2.8 MPa, these values were also similar to those in the intact model. Relative to the intact model: Group 1 showed the largest stress increase, at 26.9% (reaching 3.3 MPa); Group 2 showed a maximum stress increase of 9.7% (reaching 3.4 MPa); Group 3 showed a maximum stress increase of 9.1% (reaching 3.6 MPa).

**FIGURE 8:**
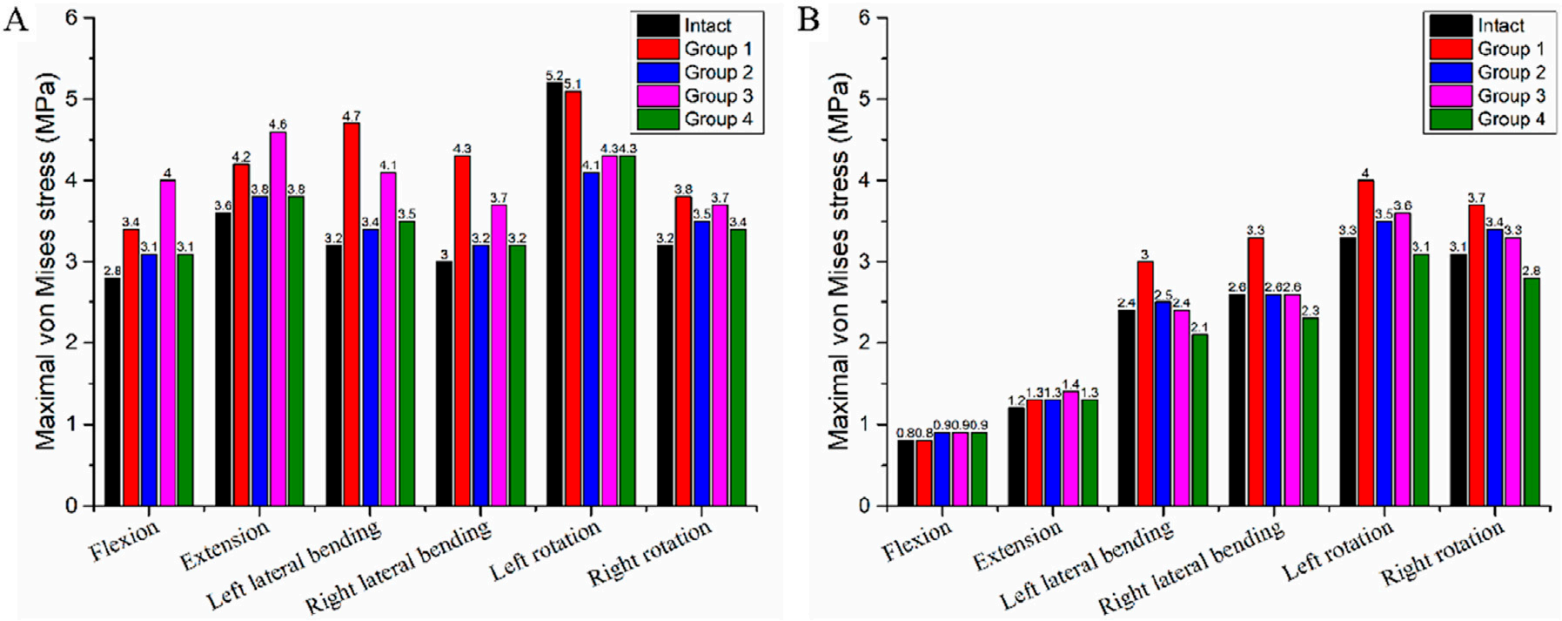
Maximum von Mises Stress of the Intervertebral Discs in the Intact Model and Four Different Internal Fixation Models. **(A)** Stress of the C3-C4 intervertebral disc; **(B)** Stress of the C7-T1 intervertebral disc.

Figure 8 illustrates the von Mises stress distribution of the C3-C4 and C7-T1 intervertebral discs across all groups and the intact model.

## 4 Discussion

ACCF, a pivotal therapeutic modality for multi-segmental cervical spine lesions, has long been a focus of clinical attention due to concerns regarding its stability and internal fixation efficacy. Currently, a diverse range of internal fixation systems has been developed for ACCF, with the classic combination of VBS, TP, and TMC serving as a representative example. Subsequently, ATPS enabled three-column fixation, which further enhanced the fixation strength of VBS (Koller et al., 2008a). With the advancement of additive manufacturing technology, the development of personalized AVB and ATPS-compatible plate designs has been achieved (Pei et al., 2022), configurations that theoretically offer superior biomechanical reconstruction performance. To date, FE analyses have been conducted to evaluate the biomechanical properties of various internal fixation systems for ACCF (Wu et al., 2017; Zhao et al., 2018; Li et al., 2022; Huang et al., 2023; Ye et al., 2024). However, these analyses have been limited to either single-segment ACCF or unilateral ATPS-TP-TMC configurations. In the present study, FE analysis was utilized to compare the biomechanical characteristics of four distinct internal fixation systems in the context of two-segment ACCF. Group 4 (4 ATPS + 3D-AVB system) provides the best surgical segment stability, minimizes stress on implants and bone-implant interfaces, protects adjacent discs, and ranks first in recommended priority for two-segment ACCF. The findings of this study provide valuable references for clinical decision-making regarding the selection of internal fixation systems.

### 4.1 ROM

Reduced ROM in the surgical segment correlates with greater stability, a higher fusion rate, and a lower risk of internal fixation loosening (Huang et al., 2023). This study demonstrated that all four internal fixation systems effectively reduced the ROM of the C4-C7 segment. Specifically, Group 4 (4 ATPS + 3D-AVB) exhibited the most significant ROM restriction across six movement directions—with a nearly 98% reduction in flexion/extension—followed sequentially by Group 3 (2 ATPS +2 VBS + 3D-AVB), Group 2 (4 VBS + 3D-AVB), and Group 1 (4 VBS + TMC). This observation can be attributed to the mechanical advantages of ATPS: an increased number of ATPS enhances the stability of the surgical segment, thereby creating a favorable microenvironment for bone fusion and ensuring long-term implant stability. In contrast, Group 1 (VBS + TP + TMC) showed the least ROM reduction (only 68.5% in flexion). This finding suggests that the traditional VBS-titanium mesh fixation may lack sufficient stability in two-segment ACCF, which helps explain the clinical occurrence of loosening or displacement in multi-segment fixation cases. Additionally, across all groups, the restriction of flexion and extension movements was more effective, whereas the restriction of lateral bending and rotational ROM was slightly less optimal. This phenomenon may be explained by the load-transfer mechanism of the graft in the reconstructed structure with anterior plating fixation: the plate-like tension band primarily restricts flexion and extension, while the graft assumes a leading role in load transfer (DiAngelo et al., 2000). This result implies that postoperative limitation of lateral bending and rotational activities may be necessary to optimize surgical outcomes.

Adjacent segment disease (ASD) refers to degeneration of segments adjacent to a fused spinal level and is a critical pre-operative consideration for cervical corpectomy patients. One study reported an 8.6% ASD incidence, with some cases requiring reoperation (Foreman et al., 2024). Compensatory increases in adjacent segment ROM accelerate disc degeneration and raise ASD risk (Wong et al., 2020), as fused segment immobility induces greater mobility in non-fused (adjacent) segments (Choi et al., 2021; Ye et al., 2024). Consistent with this, while Group 4 provided superior stability to the C4-C7 surgical segment, it induced compensatory physiological movement of the cervical spine that shifted to adjacent segments—most notably a 54.8% increase in ROM at the C3-C4 segment during extension and a 19.4% increase at the C7-T1 segment during flexion, indicating non-negligible ASD risk, especially at C3-4. It is important to note, however, that ASD is a multifactorial condition involving complex interactions between biological and mechanical factors. These factors include, but are not limited to, the natural degenerative process of the adjacent intervertebral disc and the anatomical disruption of adjacent segments induced by the initial surgical procedure (Helgeson et al., 2013).

### 4.2 Stress on implant

Elevated implant stress impairs fatigue resistance and long-term stability of internal fixation systems, thereby inducing implant fatigue fractures, fixation failure, and increased nonunion risk. In this study, Group 4 exhibited significantly lower maximum stress in its internal fixation system than the other three groups, with a peak of only 54.8 MPa under lateral bending. By comparison, Group 1, Group 2, and Group 3 showed stress values of 180.4 MPa, 255.80 MPa, and 113.50 MPa, respectively, under flexion loading. As shown in the stress cloud map, maximum stress was mainly localized at the screw-plate contact interface, consistent with previous findings (Li et al., 2022). VBS only fix the anterior and middle spinal columns; under loading, this limited fixation generates excessive lever forces, elevating screw stress. In contrast, ATPS fix the spine via bilateral pedicles, enabling more uniform stress distribution across vertebral bodies and effectively reducing local stress concentration. Additionally, 3D-AVB integration may further reduce stress. Due to its superior morphological compatibility with vertebral endplates, 3D-AVB optimizes load transfer through its porous structure (Hunn et al., 2020), avoiding the stress concentration associated with point contact in traditional TMC.

Notably, while the 3D-AVB in Group 2 improved implant-vertebral contact, the insufficient fixation strength of oblique VBS—whether superior or inferior—conversely increased screw stress. In contrast, the ATPS used in Groups 3 and 4 not only enhanced the overall fixation stability but also significantly reduced the maximum stress of the system. Collectively, these results indicate that oblique screws are mechanically infeasible for the fixation scenario investigated in this study. Similarly, Hussain et al. also found that screw divergence from the endplates not only increases load transmission to the graft but also predisposes the screws to higher shear forces after corpectomy reconstruction (Hussain et al., 2009). Specifically, Group 2 (which adopted oblique VBS) exhibited higher stress than Group 1, further confirming that oblique screw fixation is mechanically unsuitable for multi-segment ACCF procedures.

### 4.3 Stress at bone-implant interface

Implant displacement, dislodgement, and subsidence are closely associated with the bone-implant interface. Excessive vertebral endplate loading can induce implant displacement and endplate damage, ultimately leading to internal fixation failure and nonunion (Zhao et al., 2018; Shen et al., 2022). This study showed Group 4 had the lowest maximum stress at the C4 and T1 bone-implant interfaces, indicating lower fixation failure risk than other groups. In contrast, Group 2 exhibited the highest stress at these interfaces, even exceeding Group 1. This suggests that despite using endplate-matched 3D-AVB, Group 2’s obliquely inserted superior/inferior VBS damaged C4 and T1 endplates, which instead increased interface stress and implant subsidence risk. As seen in stress distribution maps, Group 2’s stress concentrated at vertebral endplate screw holes, confirming that impaired endplate integrity exacerbates implant stress concentration and failure risk. In comparison, Groups 3 and 4 had lower interface stress, with Group 4 performing best. This is directly due to ATPS’s three-column fixation advantage: ATPS not only provides higher fixation strength but also transmits loads to posterior vertebral structures via pedicles, effectively reducing stress on anterior/middle column interfaces (Huang et al., 2023). Additionally, 3D-AVB’s morphological compatibility with endplates further minimizes local interface stress concentration. Notably, Group 3’s C4 interface stress was higher than Group 4’s. This difference implies that using ATPS only in the surgical segment’s inferior vertebra may not fully optimize stress distribution, whereas bilateral ATPS application in both superior and inferior vertebrae (Group 4’s strategy) achieves more effective stress optimization.

### 4.4 Stress on intervertebral discs

Adjacent intervertebral disc stress is closely linked to ASD (Arun et al., 2009; Zhang et al., 2022), and its magnitude directly reflects the mechanical load on the disc’s annulus fibrosus and nucleus pulposus. Prolonged stress exceeding the disc’s physiological tolerance tends to induce annulus fibrosus microdamage, nucleus pulposus dehydration or herniation, and ultimately accelerate disc degeneration (Ansaripour et al., 2022). In this study, Group 4 showed a slight increase in adjacent segment (C3-C4, C7-T1) ROM, while disc stress remained close to the intact model. In contrast, other groups (e.g., Group 1) had more significant adjacent disc stress elevation—with a 46.9% rise in left lateral bending. This may be due to Group 4’s rigid fixation: though restricting surgical segment movement, it avoided excessive load transfer to adjacent segments by optimizing stress distribution. This suggests a highly stable internal fixation system may not significantly increase ASD risk if it achieves uniform stress transmission, whereas conventional systems (with insufficient stability causing abnormal movement) may exacerbate adjacent disc load. Notably, baseline stress at C3-C4 was significantly higher than at C7-T1 in the intact model. After ACCF, the procedure more prominently increased intradiscal stress in discs superior to C3-C4. Consequently, these superior discs are more prone to degeneration than inferior ones, consistent with previous findings (Fu et al., 2013; Ye et al., 2024).

### 4.5 Clinical significance

With the application of intraoperative navigation and 3D printing (Li et al., 2018; Zhang et al., 2019), the accuracy and safety of ATPS placement have been significantly improved. Additionally, previous studies on modified unilateral ATPS internal fixation systems for multi-segment ACCF have achieved satisfactory clinical and biomechanical outcomes (Zhao et al., 2011; Wang et al., 2021; Huang et al., 2023; Ye et al., 2024). Notably, only one ATPS can be inserted per vertebral level: ATPS entry points are contralateral at C3-C6 and very close to the midline at C7 (Zhao et al., 2014). Furthermore, current anterior plates are incompatible with ATPS (plate holes cannot accommodate the screws), and no clinically accepted ATPS-plate system is available for routine use. In our previous study, a custom 3D-printed bilateral transpedicular plate combined with an artificial vertebral body—designed using preoperative 3D-CT data, addressed these challenges and achieved excellent clinical results in multi-segment ACCF for cervical fractures (Pei et al., 2022). In the present biomechanical study, the 4 ATPS + 3D-AVB system exhibited the smallest range of motion (ROM) at the C4–C7 surgical segments (e.g., 97.9% reduction in flexion ROM and 98.3% reduction in extension ROM), attributed to the three-column fixation advantage of ATPS and the anatomical compatibility of 3D-AVB. Although the adjacent C3–C4 and C7–T1 segments of this group showed increased ROM (a result of compensatory adaptation due to the nearly fixed C4–C7 segments)—a phenomenon that has raised theoretical concerns about proximal junctional kyphosis angle—we further analyzed its clinical risks using adjacent intervertebral disc stress data. The results showed that the adjacent disc stress of the 4 ATPS + 3D-AVB group remained close to that of the intact model (difference <10%). Combining findings from our prior clinical research and the biomechanical results of the present study, we maintain confidence in the application of anterior surgical approaches for multi-segment ACCF.

### 4.6 Limitations

This FE study has several limitations. First, the cervical FE model does not include muscles, preventing full simulation of the intact cervical spine’s natural physiological state. Second, the model was constructed based on a single healthy individual, thus, the data may not be generalizable to the broader population, particularly patients with degenerative spinal changes, spinal trauma, or osteoporosis. Third, to ensure better FE model convergence, the screw model was simplified and lacked threads. Notably, this simplification limits the model’s representativeness, highlighting the need for further comprehensive investigations.

## 5 Conclusion

The study findings support prioritization of the four ATPS + 3D-AVB system for two-segment ACCF. Compared with the other internal fixation systems evaluated, this system offers several advantages: it provides superior stability of the operated segments, reduces stress on implants, bone-implant interfaces, and intervertebral discs, and thereby lowers the risks of implant failure and ASD. For clinical scenarios where the four ATPS + 3D-AVB system is inapplicable, the alternative options are recommended in the following priority order: two VBS + two ATPS + 3D-AVB, four oblique VBS + 3D-AVB, four VBS + TP + TMC. Future clinical trials are required to verify the long-term efficacy and safety of these internal fixation systems in clinical practice.

## Data Availability

The original contributions presented in the study are included in the article/Supplementary Material, further inquiries can be directed to the corresponding authors.
