# Supplementary material for: Biomechanical evaluation of four internal fixation systems for two-segment anterior cervical corpectomy and fusion: a finite element analysis
Source: Front Bioeng Biotechnol. 2025 Oct 28;13:1691524. doi: 10.3389/fbioe.2025.1691524 (PMC12602490; doi:10.3389/fbioe.2025.1691524)
Supplement: Supplementary file 1 [file DataSheet1.pdf]

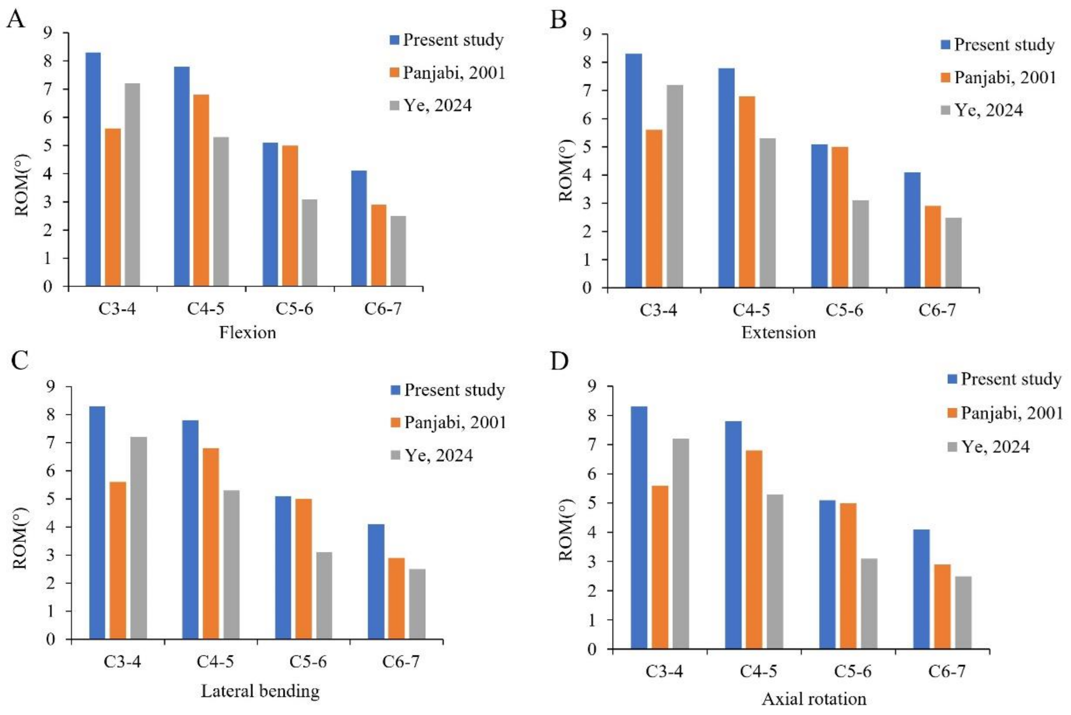

Supplement Figure 1. Comparison of the ROM of the FE model of C3-C7 with the previous biomechanical studies. A. ROM in flexion, B. ROM in extension, C. ROM in lateral bending, D. ROM in axial rotation.
